# Did homeobox gene duplications contribute to the Cambrian explosion?

**DOI:** 10.1186/s40851-014-0004-x

**Published:** 2015-01-13

**Authors:** Peter W H Holland

**Affiliations:** Department of Zoology, University of Oxford, South Parks Road, Oxford, OX1 3PS UK

**Keywords:** Evolution, Gut, Embryonic development, Bilateria, ParaHox, Hox, NK gene, Burrowing

## Abstract

The Cambrian explosion describes an apparently rapid increase in the diversity of bilaterian animals around 540–515 million years ago. Bilaterian animals explore the world in three-dimensions deploying forward-facing sense organs, a brain, and an anterior mouth; they possess muscle blocks enabling efficient crawling and burrowing in sediments, and they typically have an efficient ‘through-gut’ with separate mouth and anus to process bulk food and eject waste, even when burrowing in sediment. A variety of ecological, environmental, genetic, and developmental factors have been proposed as possible triggers and correlates of the Cambrian explosion, and it is likely that a combination of factors were involved. Here, I focus on a set of developmental genetic changes and propose these are part of the mix of permissive factors. I describe how ANTP-class homeobox genes, which encode transcription factors involved in body patterning, increased in number in the bilaterian stem lineage and earlier. These gene duplications generated a large array of ANTP class genes, including three distinct gene clusters called NK, Hox, and ParaHox. Comparative data supports the idea that NK genes were deployed primarily to pattern the bilaterian mesoderm, Hox genes coded position along the central nervous system, and ParaHox genes most likely originally specified the mouth, midgut, and anus of the newly evolved through-gut. It is proposed that diversification of ANTP class genes played a role in the Cambrian explosion by contributing to the patterning systems used to build animal bodies capable of high-energy directed locomotion, including active burrowing.

## Review

### Introduction

The number, diversity, and disparity of fossilized animal forms are far greater in deposits dating to the early Cambrian [1] than found in any earlier deposits. Although some have claimed that this difference reflects fossilization bias, the majority view is that most of the diversity of animal life increased in the early Cambrian, a phenomenon called the Cambrian explosion. As recently reviewed [1] a multitude of factors have been proposed as candidates for triggering the observed diversification, including biotic and abiotic factors. These authors conclude that was not likely a single cause, but rather a cascade of interconnected environmental and ecological changes, including sea level rise generating habitable shallow-water seas, submarine erosion releasing calcium and phosphate, and exploitation of calcium by animals for biomineralization.

When considering this multitude of factors that contributed to the diversification of animals, internal constraints should not be ignored. Unless the appropriate genes, and their associated developmental pathways, are in place to build complex and adaptable body plans, no combination of environmental factors alone could have stimulated the radiation of the animal kingdom. Based on discovery of the ParaHox gene cluster (a set of three genes related to the better known Hox genes), I previously proposed that generation of these new Hox-related homeobox genes in early animal evolution permitted elaboration of the bilaterian body, especially in allowing evolution of distinct mouth and anus, and that this was instrumental to the diversification of animal life in the Cambrian. We suggested that “*the origin of distinct Hox and ParaHox genes by gene-cluster duplication facilitated an increase in body complexity during the Cambrian explosion”* [2]. This idea was expanded more generally, ‘*the origin of three germ-layers, bilateral symmetry and a through gut also probably involved gene duplication….. This event may provide a partial genetic explanation for the Cambrian explosion*’ [3]. The relevance of the through-gut to the Cambrian explosion, but without reference homeobox genes, was also later stressed by Cavalier-Smith [4] who wrote *“Very likely this anal breakthrough …. stimulated the long-puzzling Cambrian explosion”.*

Here I revisit this hypothesis, explaining the basis for its proposal and evaluating its robustness in the light of additional data accumulated over the past 15 years. I conclude that there was indeed an expansion of the ANTP class of homeobox genes during early animal evolution, but on a larger scale than originally thought. This large expansion generated not only the Hox and ParaHox genes, but also the NK homeobox genes and their relatives, and I suggest that these genes were recruited for roles in patterning ectoderm, gut and mesoderm. These patterning genes, and others, permitted the evolution of animal body plans capable of active, directed locomotion and, due to evolution of a through-gut with a distinct anus, active burrowing and feeding in sediments. These internal factors acted alongside, or prior to, external stimuli to catalyse the Cambrian explosion.

### What exploded in the Cambrian?

The fossils of the Cambrian record a diversification of animal life, but how does this relate to the phylogeny or evolutionary tree of the animal kingdom? In other words, which clade or clades of animals actually diversified? In 1857, Darwin wrote to his friend Thomas Henry Huxley *“The time will come, I believe, though I shall not live to see it, when we shall have very fairly true genealogical trees of each great kingdom of nature”* [5]. Darwin did not live to see it, and indeed reconstruction of the *‘fairly true’* outline of metazoan phylogeny was not achieved until the late 1990s [6,7]. After more than a century of argument, there is remarkable consensus today about the general framework of animal phylogeny: a framework originally built from ribosomal DNA sequences, but now corroborated by DNA sequences from hundreds of genes [8-10]. The broadly-accepted scheme, sometimes called the ‘New Animal Phylogeny’, sees four animal phyla branching off basally (Porifera, Ctenophora, Placozoa, Cnidaria – not necessarily in that order). All animals apart from the basal four phyla belong to the Bilateria, also called triploblasts, which can be divided into three great clades: the Ecdysozoa (moulting animals, including arthropods, onychophorans, tardigrades, nematodes, priapulids, and others), the Lophotrochozoa (including molluscs, annelids, platyhelminths, nemerteans, brachiopods, bryozoans, and more) and the Deuterostomes (chordates, echinoderms, hemichordates).

The bilaterians are characterized, at least primitively, by possession of an anteroposterior axis (head to tail), a dorsoventral axis (top to bottom) and a left-right axis. Most bilaterians have a centralized nerve cord with a brain, and sense organs concentrated at a clear front end. They (primitively) have a through-gut with two openings, ingesting food through a mouth and passing food unidirectionally before ejection of waste through an anus. Either side of the gut are muscle blocks capable of contorting the body, often acting antagonistically to hydrostatic skeletons or hard parts. These characters are in sharp contrast to the four basal phyla, none of which have cephalization, brains, centralized nerve cords, or through-guts. The cnidarians, for example, have a single gastric opening which acts as both mouth and anus (though there are some variations on the theme). In general, the bilaterians are animals characterized by very active locomotion in a clear direction. These are the animals that explore the world in three-dimensions with forward-facing sense organs, a brain, and an anterior mouth, that possess muscle blocks to allow crawling, burrowing or even swimming, and that have an efficient gut system to process food and eject waste in the wake of the moving animal. Some cnidarians also burrow, but due to the absence of an anus and defined muscle blocks, their burrowing is not in such a directed, efficient, or high-energy manner. The bilaterians are truly the animals that dominate the three-dimensional world (for further discussion see [11]).

Over twenty years ago, Conway Morris argued that the Cambrian explosion was essentially a bilaterian phenomenon [12]. Most Ediacaran fossils can be assigned to a ‘grade’ of organisation comparable with that found in cnidarians, or if they were not cnidarians then they were also not clearly bilaterians either. In contrast, the majority of animals that characterise the Burgess Shale-type deposits of the middle Cambrian, or the earlier small shelly faunas, are bilaterian. Details of this idea have been challenged several times, but overall it holds considerable weight. For example, comparisons between living animals indicate that the latest common ancestor of all living bilaterians (LCAB) had a brain, anterior sense organs, lateral muscle blocks, central nervous system, and a through-gut [13,14]. An animal with such a body organisation should be capable of active directed locomotion, including burrowing, and logically such animals should leave traces in the form of burrows and directional tracks. Putative trace fossils have been reported from 560 million years ago, 20 million years before the Cambrian, but the clearest of these are surface traces; the first complex horizontal and vertical burrows date to the base of the Cambrian itself [15,16]. One would also deduce that the first bilaterians were not particularly small, since today the microscopic meiofaunal animals are secondarily derived from larger animals, and are highly modified. Furthermore, comparison of developmental genes involved in mesoderm and heart development suggest that the LCAB had a circulatory system [17], which is not compatible with that ancestor being a microscopic organism. The LCAB should, therefore, have been at least several millimetres – perhaps tens of millimetres – in size. Hence, the trace fossil record should be a very good indicator of whether bilaterians, or at least bilateral animals that postdate the LCAB, were present.

Putting the fossil evidence (including trace fossil evidence) together with comparative anatomy and developmental biology, I argue that the latest common ancestor of living bilaterians dates to the base of the Cambrian and not significantly before. There are lines of evidence that dissent from this view, notably dating efforts using molecular clocks that have placed the LCAB much earlier [18,19]. Such analyses are highly sophisticated, but since we do not fully understand the factors that dictate mutation and substitution rates in genes, one has to ask if this evidence is strong enough to challenge the robust paleontological record. On balance, I favour the view that the Cambrian explosion represents a true diversification of the Bilateria, from a LCAB that existed at or only marginally before the base of the Cambrian. An alternative view would place the LCAB (with its large size, circulatory system, central nervous system, muscles, and through-gut) rather earlier, and the Cambrian explosion would represent diversification of descendent lineages.

### The anus of fortune

The earliest stage of the Cambrian is the Fortunian, named after the town of Fortune in Newfoundland, Canada. Deposits dating to this period, around 540–530 million years ago, show trace fossils including indications of animals that had directed locomotion, sinusoidal movement and some capacity for burrowing, including the diagnostic mud-burrowing trace fossil *Trichophycus pedum* [16,20]. It is thought that animal movement and burrowing caused break-up of the dominant microbial mat faunas, mixing of anoxic and oxic layers of sediments, and an increase in the habitable zone by animals. We may ask which of the bilaterian characters were instrumental in permitting these new forms of animal behaviour? Furthermore, which genes or developmental processes were necessary to permit these characters to evolve? There are living cnidarians that burrow, but these are mostly restricted to very soft mud and they do not mix sedimentary layers extensively. In contrast, many bilaterian ‘worms’ dig, delve, and devour their way through compacted sediments the world over.

The efficiency of bilaterian burrowing rests on three key features: muscle, skeletons, and a through-gut. Muscles provide the power force necessary for high-energy burrowing, and skeletons (which may be composed of hard parts or more usually of fluid-filled cavities) provide incompressible structures ensuring that muscular forces do not simply make bodies shrink but transmit energy against the surrounding substrate. Through-guts allow feeding to be combined with locomotion through sediments. Animals with these characters can truly explore their world in three dimensions, leaving their waste products behind. A centralised nervous system with anterior brain and sense organs to face the on-coming world is a logical set of adaptations to complement this body plan.

As for intrinsic factors necessary for animals to develop these characters, the key must be sets of genes that control spatial patterning of mesoderm (including muscle), endoderm (forming most of the gut) and ectoderm (including the nervous system). There are many candidates for such genes, including those encoding transcription factors from the Fox, Pax, homeobox, zinc finger and T-box superclasses and also genes coding for secreted molecules or their receptors. In this article I focus on homeobox genes [21], but by doing so I am not suggesting that other types of genes are less important. Indeed, it is likely that complex networks of genes needed to be pieced together during evolution to allow development of particular features. It is also worth noting that it is not necessarily the origin of the genes themselves that is the key permissive step for evolution of a particular anatomical feature, but rather it could be the co-option of those genes into regulatory networks associated with a function. In other words, it is not the birth of the genes that matters, but their deployment. With this background in mind, I will summarize recent data concerning the evolution of homeobox gene functions and their association with patterning of mesoderm, ectoderm and endoderm, including genes for specifying the mouth, central gut, and anus of the bilaterian through-gut.

### The ANTP homeobox genes: Hox, ParaHox, NK and relatives

When homeobox genes are mentioned, many biologists will think of Hox genes – a well known set of genes famous for their roles in body patterning and their astonishing evolutionary conservation. First discovered in the fruit fly *Drosophila*, mutated Hox mutated typically cause ‘homeotic’ transformations, when one part of the body is transformed into another, such as legs growing where antennae should be. In many animals these genes are arranged in ‘gene clusters’, meaning that Hox genes are neighbours on a chromosome. This in itself is quite unusual and central to how Hox genes are regulated. But Hox genes are just one type of homeobox gene. They are the tip of the homeobox iceberg [21]. For example, *Drosophila* has over a hundred homeobox genes, yet only eight of these are typical Hox genes. Similarly, the human genome has over 200 homeobox loci, of which just 39 are Hox genes [22,23]. To make sense of the diversity of homeobox genes, they can be subdivided into gene classes and gene families. Of the 11 homeobox gene classes in animals, the one that contains most of the genes implicated in body patterning is the ANTP class. This contains the Hox genes, but also some other closely related homeobox genes including ParaHox genes, NK genes and various others (notably Barhl, Barx, Bsx, Dbx, Dlx, Emx, En, Evx, Gbx, Hhex, Hlx, Meox, Mnx, Msx, Nanog, Noto, Vax and Ventx [24]).

The ParaHox genes are particularly intriguing. It was noted in the 1990s that some homeobox genes that are not part of Hox gene clusters are especially similar in DNA sequence to Hox genes, and for some time their evolutionary origins remained unclear. Indeed, for three of these genes – Gsx, Xlox (also called Pdx) and Cdx – their DNA sequences are more similar to some Hox genes than many Hox genes are to each other. They are ‘Hox-like,’ but not in the Hox cluster. The paradox was solved, or at least clarified, in 1998 when we found that these three genes were arranged in their own gene cluster, at least in one animal taxon – the cephalochordate amphioxus [2]. Subsequently, clustering of these three genes has also been found in vertebrates (except teleost fish [25]) and in an echinoderm, the sea star *Patiria miniata* [26]. The discovery of the gene cluster in amphioxus, together with phylogenetic analysis of Hox and ParaHox sequences, led to our proposal that Hox and ParaHox gene clusters were generated by an ancient gene cluster duplication event, from a hypothetical progenitor ‘ProtoHox’ gene cluster. Further studies in other taxa have added support to this model, with the main issue of contention simply being whether the hypothetical ProtoHox gene cluster had two, three or four homeobox genes [27,28]. Not all animals have retained the ParaHox genes in a clustered arrangement, and some taxa have lost particular ParaHox genes, but the original bilaterians must have possessed an intact ParaHox gene cluster. There was also confusion for some years over the timing of the deduced Hox-ParaHox duplication event, but it is now clear that it was not an event that characterised the bilaterians uniquely, since it occurred before the divergence of cnidarians and bilaterians [29]. The implication is that the origin of Hox genes and the simultaneous origin of ParaHox genes occurred before the origin of the bilaterian body plan.

**Figure 1:**
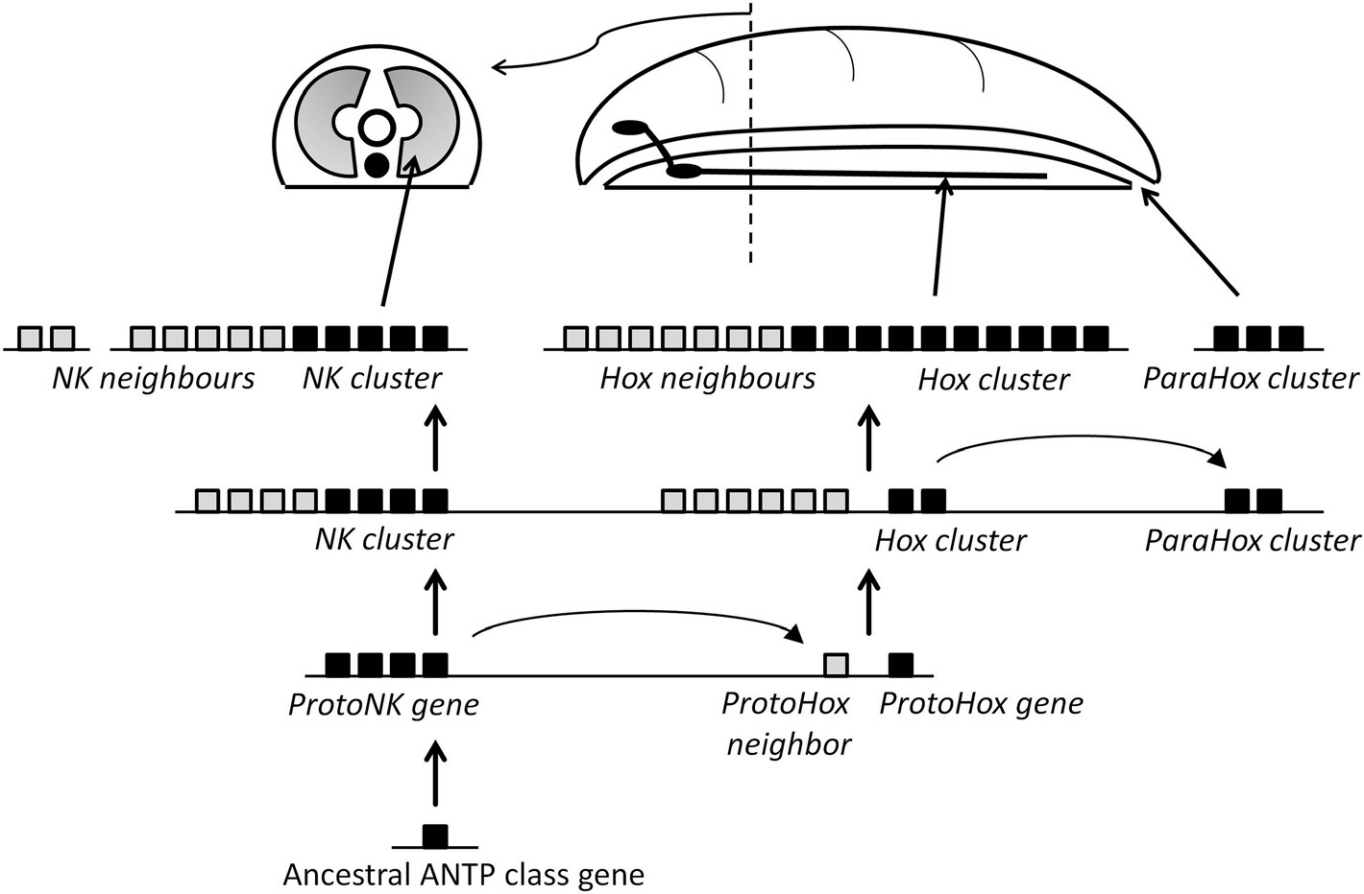
**Tandem homeobox gene duplication and recruitment to body patterning.** Tandem duplication of homeobox genes in early metazoan evolution generated ProtoNK and ProtoHox genes (black boxes), plus neighbouring homeobox genes (grey boxes). The ProtoHox gene cluster then duplicated to generate Hox and ParaHox gene clusters. It is proposed that the NK gene cluster, Hox gene cluster and ParaHox gene cluster were recruited to pattern the mesoderm, ectoderm (including central nervous system) and through-gut of the first bilaterian animals. Adapted from [21].

What these findings mean for the evolution of body patterning, and indeed the Cambrian explosion, must also take into account another key set of ANTP class homeobox genes, in another gene cluster: the NK homeobox genes. These genes have been studied in most detail in the fruit fly, *Drosophila*, where they form a third and quite separate gene cluster, containing the genes *slo* (*NK1*), *tin* (*NK4*), *bap* (*NK3*), *Lbx* and *Tlx* [30]. All five genes are ancient and date to the base of the Bilateria, or indeed earlier, and are well conserved in a range of taxa. Although the NK gene cluster has broken up in amphioxus and vertebrates of the Phylum Chordata [31], there is evidence from comparison of many genomes that the five-gene cluster found in *Drosophila* is a remnant of an even larger gene cluster. The ancestral bilaterian NK gene cluster most likely contained the five homeobox genes noted above, plus several others including *Msx*, *NK5* and *NK6* [21,32-34]. Phylogenetic analysis reveals that the Hox and ParaHox are more closely related to each other than they are to the NK cluster. Sponges have several NK homeobox genes but not definite Hox or ParaHox genes [35], and it has been suggested that Hox and ParaHox genes were generated from an ancestral NK-like cluster by tandem gene duplication (Figure 1).

To summarize, before the emergence of bilaterian animals, three distinct clusters of ANTP class homeobox genes arose: Hox, ParaHox and NK homeobox genes. Additional ANTP class genes were left scattered as neighbours of the Hox and NK homeobox genes. The simplest model to explain the origins of all these genes, now supported by a large amount of data from diverse taxa, is that a hypothetical ancestral ANTP class gene underwent extensive tandem gene duplication, spreading a series of related genes along a chromosome (Figure 1). From this large array of genes, many genes subsequently became dispersed in the genome, but there remained three ‘islands’ of gene clustering - NK, Hox and ParaHox - each with a subtly different developmental role, discussed below. The three gene clusters have been conserved to different extents in different evolutionary lineages. To take two extremes, vertebrates have very compact and conserved Hox and ParaHox gene clusters but disrupted NK gene clusters, while dipteran flies have a disrupted Hox gene cluster and have lost one of the three ParaHox genes, but have retained a tight NK gene cluster.

### The germ-layer hypothesis

The presence of three ANTP class gene clusters has an intriguing parallel to the three germ layers of bilaterian animals. This relationship had been outlined previously [21] and is expanded below. The Hox genes of vertebrates and the fruit fly, the first to be analysed, are deployed to pattern the embryonic mesoderm and ectoderm (especially neuroectoderm). In mammals, for example, mutation of Hox genes causes profound disruption to patterning of the vertebral column, which develops from the mesodermal somites, and also the nerve cord. From these data alone, it might reasonably be thought that, in the common ancestor of insects and vertebrates, Hox genes patterned ectoderm and mesoderm. However, early studies on amphioxus and ascidians reviewed by [36], and later work on molluscs and annelids [37,38], has highlighted that the most consistent picture is for Hox genes to have anteroposterior spatial patterns in ectoderm only. Mesoderm expression, when present, rarely shows a clear anteroposterior domain, except in vertebrates and insects. Perhaps the original role of Hox genes in Bilateria, therefore, was to encode positional information just in the ectoderm? Interestingly, the Hox genes do not pattern the most anterior parts of the brain, but other homeobox genes originally linked chromosomally to Hox genes (such as *Emx*, *En* and *Gbx*) are deployed in these regions.

**Figure 2:**
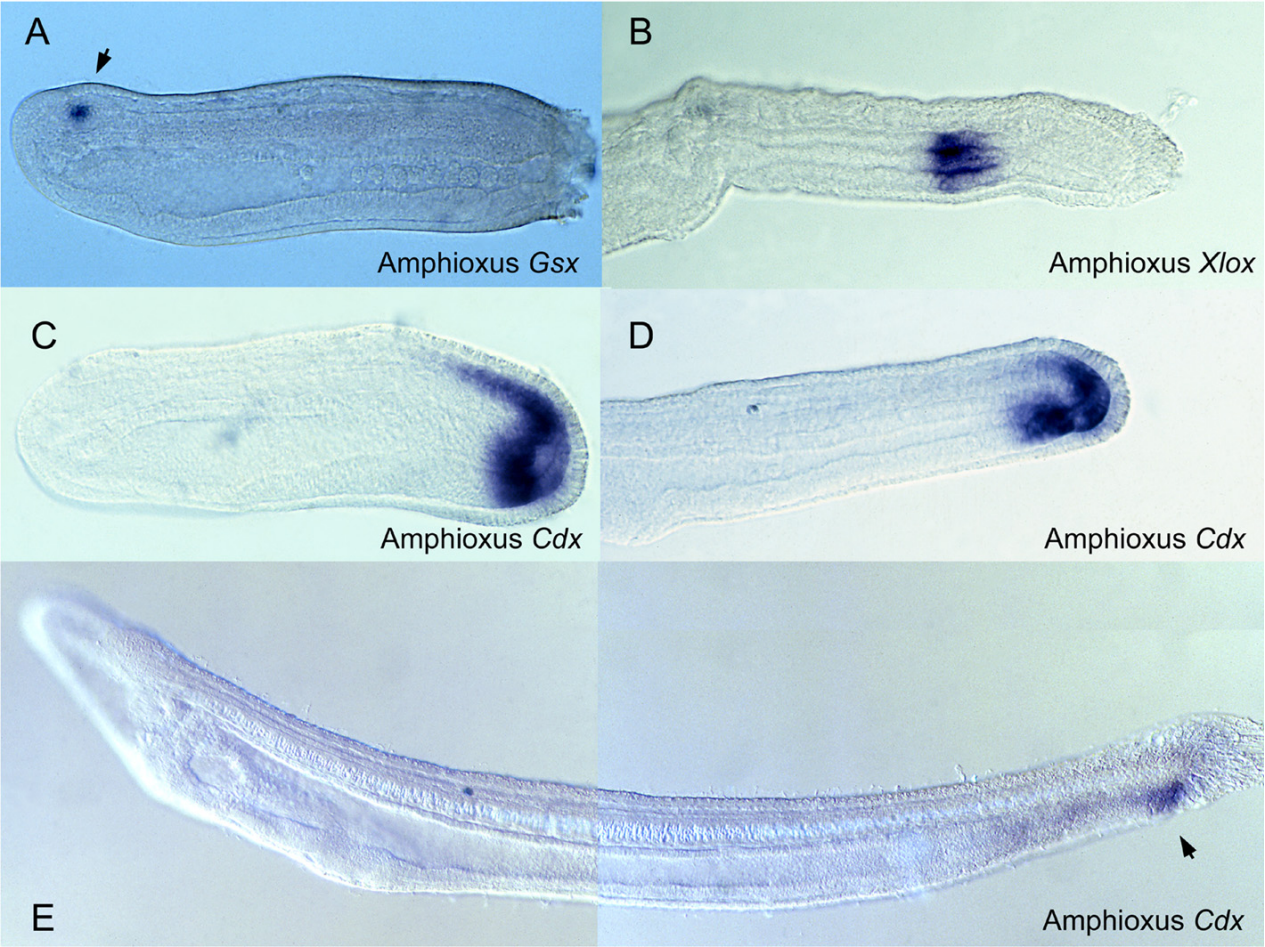
**Expression of ParaHox genes in embryos and larvae of amphioxus** ***Branchiostoma floridae*** **.** Whole mount in situ hybridisation used digoxigenin-labelled riboprobes and expression is visualised as blue stain. Each photograph is a lateral view with anterior to the left and dorsal to the top. **(A)** Gsx gene expression in a neural structure, the cerebral vesicle (arrowhead); ~20 hour embryo. **(B)** Xlox gene expression in midgut endoderm; ~36 hour larva. **(C)** and **(D)** Cdx gene expression in the hindgut and posterior neural tube of ~20 hour embryo and ~30 hour embryo respectively. **(E)** Cdx gene expression around the anus of 2.5 day larva (arrowhead).

**Figure 3:**
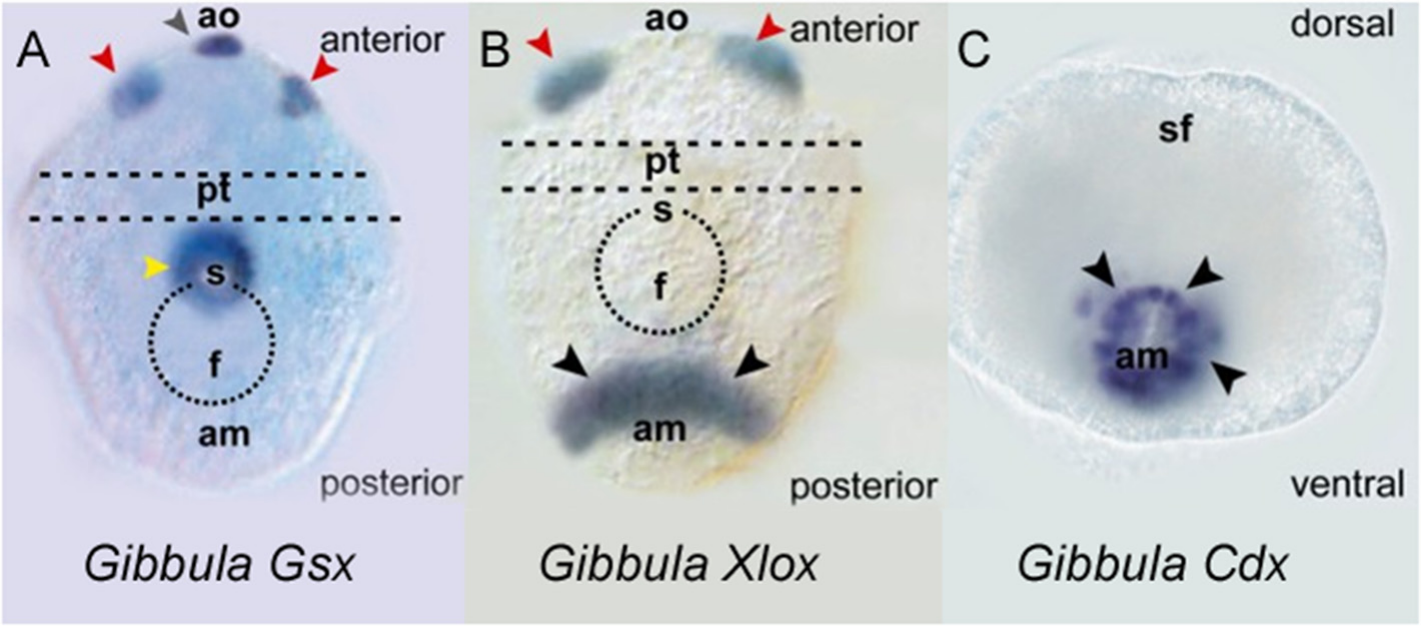
**Expression of ParaHox genes in trochophore larva of a mollusc** ***Gibbula varia*** **.** Gene expression is visualised as blue stain. **(A)** Gsx gene expression in stomodeum or mouth (yellow arrowhead) plus the apical sensory organ (black arrowhead) and two groups of cells marking the developing cerebral ganglion (red arrowheads). **(B)** Xlox gene expression in a semicircle anterior to the anal region (black arrowheads) and in two groups of anterior cells (red arrowheads). **(C)** Cdx gene expression in a ring around the anal marker (black arrowheads); posterior view. am anal marker, ao apical organ, f foot rudiment, pt prototroch, s stomodeum, sf shell field. Reproduced from [43]; original publisher BioMed Central.

With the discovery of the ParaHox gene cluster in amphioxus, an intriguing parallel was found. Two of the ParaHox cluster genes, *Cdx* and *Xlox* (*Pdx*) were found to be predominantly expressed in the gut [2]. *Cdx* is consistently expressed in the most posterior part of the body, around the anus and also in other tissues, in all bilaterian animals examined (Figures 2 and 3). *Xlox* is necessary for development of the endoderm of the presumptive pancreas and duodenum of vertebrates [39,40], and is expressed in a region of midgut endoderm in amphioxus (Figure 2) and leeches [2,41]. The same has also been shown recently for an echinoderm [26]. We proposed, therefore, that after duplication of the hypothetical ProtoHox gene cluster to generate Hox and ParaHox, one gene cluster was deployed to pattern ectoderm and the other to pattern endoderm (or more accurately gut, since the anterior and posterior extremities of the gut are not generally considered endoderm [2]). There were, however, two complications to this model. First, while there are many Hox genes in most bilaterians (around 10 being normal for an invertebrate), there are only three ParaHox genes leaving little scope for regionalising the entire gut. Second, while the *Cdx* gene is associated with the anus and *Xlox* associated with the midgut, the third ParaHox gene (*Gsx*) was not expressed in the mouth in amphioxus [2]. Instead, this gene was found to be expressed in the amphioxus brain, as indeed are its two homologues in vertebrates (Figure 2). Our proposal to cope with this uncooperative fact was that gene expression had changed in the ancestry of amphioxus and vertebrates [2]. This suggestion was expanded upon later, as follows. For over a century there had been a suggestion that deuterostomes (the clade to which amphioxus and vertebrate belong) have invented a ‘new mouth’ during evolution; if true, then lack of expression in the forming mouth of amphioxus and vertebrates might have no bearing on the ancestral role [42].

The testable prediction was that analysis of the *Gsx* gene in protostome taxa - animals which are proposed to have retained the original mouth opening - should reveal expression in the oral region. This was not the case for the fruit fly, *Drosophila*, and the gene has been lost in the nematode *Caenorhabditis*, but both these taxa have many derived features of their development. It was therefore very informative when Gsx expression was studied carefully in a spiralian protostome (the mollusc *Gibbula*), since a clear ring of expression was seen around the oral cavity, albeit alongside expression in the nervous system [43] (Figure 3). The authors noted *‘Our results support Holland’s hypothesis that* ParaHox *genes are involved in gut regionalization and offer further support to the ancestral mouth patterning role of* Gsx *in protostomes’* [43]. Perhaps the original role of ParaHox genes in Bilateria, therefore, was to encode positional information in the gut?

Above I noted that Hox and ParaHox are retained clusters from a larger array of ANTP class homeobox genes, alongside a third gene cluster, NK. The expression and function of these genes have been less well-studied, but there are indications that mesodermal expression is a common property [30]. For example, the *NK4* or *tinman* (*tin*) gene of *Drosophila* is first expressed in all mesoderm cells and later becomes restricted to the dorsal region of the mesoderm. The key role is in development of the insect pulsatile vesicle (or ‘heart’) derived from these cells; the name *tinman* refers to the heart-deficient mutant fly, named after a character in the Wizard of Oz [44]. In vertebrates there are usually three vertebrate orthologues of NK4, called *Nkx2-3*, *Nkx2-5* and *Nkx2-6*; these are expressed in the vertebrate heart in at least some species, although there may be functional overlap [45]. *Nkx2-5* in particular is necessary for correct heart development and mutations in this gene are associated with heart defects in humans [46]. The *NK3* or *bagpipe* gene is involved in *Drosophila* visceral mesoderm development [47], and its vertebrate orthologues are expressed in heart, plus other tissues including salivary and prostate glands and developing somites [48-50]. The *NK1* or *slouch* gene has roles in insect somatic muscle development and is also expressed in the nervous system [30,51]. The mouse homologues *Nkx1-1* (also called *Sax2*) and *Nkx1-2* (*Sax1*) break the pattern of mesodermal function, being predominantly expressed in neural tissue [52,53], with *Nkx1-2* in the brain and implicated in hormonal control of food intake [53]. The general trend of mesodermal expression and function, however, continues to some genes no longer in the NK gene cluster of *Drosophila*, but deduced to have been present in the original NK gene cluster of the basal bilaterian, such as *Msx* (muscle segment homeobox). Although there is no suggestion of anteroposterior restriction, in contrast to Hox and ParaHox, it seems clear that NK homeobox genes are primarily expressed in subsets of the mesoderm. There are additional sites of expression, and many non-mesodermal functions, but on balance it seems likely that the original role of NK homeobox genes was connected with specialisation of mesoderm.

## Conclusions

The base of the Cambrian was marked by the evolution of active, efficient, burrowing, and directed locomotion along and beneath the substrate. This in turn transformed an essentially two-dimensional world, dominated by microbial mats, into a three-dimensional world. It is argued here that for the evolution of this ‘ecological engineering’ , the early bilaterians required muscle, skeletons, a through-gut, and a centralised nervous system with anterior brain and sense organs. The through-gut, with distinct mouth, midgut, and anus is highlighted here as particularly important for ecological change. The developmental genetic evidence, together with comparative anatomy, indicates that these key anatomical characters were all in place in the most recent common ancestor of living bilaterians, and hence must have evolved on the bilaterian stem lineage, not independently in different bilaterians.

How did these anatomical characters, so pivotal for the Cambrian explosion, arise in evolution? The expansion of the ANTP class of homeobox genes has been traced, and is shown to have generated an array of regulatory factors, containing three key gene clusters: Hox, NK and ParaHox. The proposal, which has been developed gradually over the past 15 years, is that these three sets of new genes were recruited in evolution for patterning the development of the nervous system, the development of muscle and development of a through-gut in the ancestors of bilaterians, thereby permitting the evolution of animals capable of active directed locomotion, including burrowing through the substrate. Without this molecular evolution, there could be no Cambrian explosion. Even when environmental changes, such as sea level changes occurred, exploitation of new niches required the genetic machinery to build complex body plans.

It should be highlighted that the ANTP class homeobox genes form just part of the genetic architecture used for patterning these structures, and that many other genes were also involved. These include the Pax genes, some of which are involved in mediolateral specialisation of nervous system and mesoderm, and in formation of sense organs, the Fox genes implicated in endoderm and mesoderm patterning, the T-box genes and more [54-58]. Ultimately, without the diversity of transcription factor genes generated in early animal evolution, it is argued that no bilaterian evolution was possible.
